# Upscaling Cement Paste Microstructure to Obtain the Fracture, Shear, and Elastic Concrete Mechanical LDPM Parameters

**DOI:** 10.3390/ma10030242

**Published:** 2017-02-28

**Authors:** Gili Sherzer, Peng Gao, Erik Schlangen, Guang Ye, Erez Gal

**Affiliations:** 1 Department of Structural Engineering, Ben-Gurion University of the Negev, 84105 Beer-Sheva, Israel; sherzerg@gmail.com; 2 Microlab/Section of Materials and Environment, Faculty of Civil Engineering and Geosciences, Delft University of Technology, 2628 CN Delft, The Netherlands; P.Gao@tudelft.nl (P.G.); Erik.Schlangen@tudelft.nl (E.S.); G.Ye@tudelft.nl (G.Y.); 3 School of Materials Science and Engineering, South China University of Technology, Guangzhou 510640, China

**Keywords:** concrete material, numerical simulation, lattice model, discrete models, fracture, upscaling procedure, homogenization

## Abstract

Modeling the complex behavior of concrete for a specific mixture is a challenging task, as it requires bridging the cement scale and the concrete scale. We describe a multiscale analysis procedure for the modeling of concrete structures, in which material properties at the macro scale are evaluated based on lower scales. Concrete may be viewed over a range of scale sizes, from the atomic scale (10^−10^ m), which is characterized by the behavior of crystalline particles of hydrated Portland cement, to the macroscopic scale (10 m). The proposed multiscale framework is based on several models, including chemical analysis at the cement paste scale, a mechanical lattice model at the cement and mortar scales, geometrical aggregate distribution models at the mortar scale, and the Lattice Discrete Particle Model (LDPM) at the concrete scale. The analysis procedure starts from a known chemical and mechanical set of parameters of the cement paste, which are then used to evaluate the mechanical properties of the LDPM concrete parameters for the fracture, shear, and elastic responses of the concrete. Although a macroscopic validation study of this procedure is presented, future research should include a comparison to additional experiments in each scale.

## 1. Introduction

Modeling the behavior of concrete structures is a challenging research task, since the various significant physical phenomena are related to various spatial scales, from the cement paste scale up to the structure scale. Usually, the mechanical behavior of concrete is modeled macroscopically via plastic and/or damage constitutive relations (e.g., [1,2,3,4,5,6,7,8]) These macroscopic models are often characterized by numerous parameters that need to be calibrated so as to analyze the complex behavior of the concrete at the different stages of loading and damage modes. Since concrete comprises a considerable number of different microstructures, developing an alternative approach to macroscopic modeling is essential. Variations in the micro- to meso structures of concrete may include the addition of fibers made up of different materials; different sizes, shapes, and types of sand and aggregate; different water-to-cement ratios; and variations in the composition of the cement. Although a multiscale analysis of concrete structures is used, the development of down/up scaling formulations is required, especially in the nonlinear region of the structure response to fracture, friction, plasticity, etc. For example, several studies [9,10,11,12,13,14,15,16,17,18,19,20,21,22] developed a nonlinear meso scale model but did not deal with the down/up scaling issue, while others [23,24,25,26] developed an upscaling procedure but did not apply it to the nonlinear region. The main motivation for developing the upscaling methodology suggested in the current paper is the potential to elucidate the impact of the chemical composition of cement on the mechanical behavior of concrete, and to reduce the experimental efforts required for calibrating the parameters of the Lattice Discrete Particle Model (LDPM), as presented, for instance, in [17].

The microstructure of a cement paste can be imaged either experimentally [27] or numerically [28,29,30,31,32]. Experimentally, X-ray computed microtomography (CT) [27] can be used to collect microstructure information on cement paste in a non-destructive manner by way of digitized voxels. Numerically, models of cement hydration and microstructure formation processes were described previously [28,30,31,32]. The goal of this study is to bridge the microscopic cement paste scale and the concrete meso scale by upscaling the mechanical properties of the cement paste to obtain the LDPM concrete parameters. The proposed methodology is based on the following models: (1) The HYMOSTRUC3D model [28,30], which is capable of elucidating the mineralogical composition of cement clinkers and the chemical reaction of the cement paste; (2) the Anm material model [33], which is used to generate the unit cell geometries, including irregular shaped aggregates, for the mortar scales; and (3) the lattice model suggested by [34], which was chosen for its ability to simulate fracture processes at the cement paste and mortar scales. The LDPM was chosen to represent the meso scale behavior of concrete because it can take into account the concrete macroscopic size and shape effects.

The HYMOSTRUC3D numerical model [28,30] simulates both the reaction process and the formation of the microstructure in hydrating Portland cement—taking into account hydration kinetics and simulating the cement particles as spheres that develop during the hydration process. It is designed as a three-dimensional model, which is defined by multiple input parameters, including the water-to-cement ratio, the mineralogical composition of the cement, the size distribution of the cement particles (usually employing the Rosion–Rammler sieve curve [35]), the curing temperature, and the specimen geometry [36]. This model regards the hydration process and the microstructural formation of the cement paste.

The LDPM [16,17], numerical mechanical model, simulates the meso structure of concrete by a three-dimensional assemblage of particles that are generated randomly according to a given grain size distribution (which is usually determined using the Fuller curve approach [37]). The LDPM has been extensively calibrated and validated [17], and its superior functionality in reproducing and predicting concrete behavior under a wide range of loading conditions has been reported both qualitatively and quantitatively [16,17,18]. The LDPM can be used to simulate the important mechanical response of concrete structures, including unconfined and confined uniaxial compression tests (considering also the effect of specimen slenderness and of loading platens friction); biaxial compression tests; triaxial compression tests with a reversal of softening into hardening for increasing confinement; hydrostatic compression tests that show no softening whatsoever; direct tensile tests; splitting tensile (Brazilian) tests; fracture tests; energetic size effects; cycle loading; the correct ratio between compressive strength and tensile strength; and the rate effect on concrete strength. The interaction between the various aggregates in the concrete is taken into account at the concrete scale, and the geometry of the elements is obtained by using the Delaunay triangulation, which provides volume subdivision by tetrahedrons. The mechanical interaction between adjacent aggregate particles through the embedding mortar is represented by the mechanical response of the elements, in which each element is characterized by the aggregate embedded in the mortar. Such interactions are governed by meso scale constitutive equations, which describe the tensile fracturing with strain-softening, cohesive, and frictional shearing, and the nonlinear compressive behavior with strain-hardening. The boundaries between two elements are represented by the outward triangular faces, called facets.

**Figure 1:**
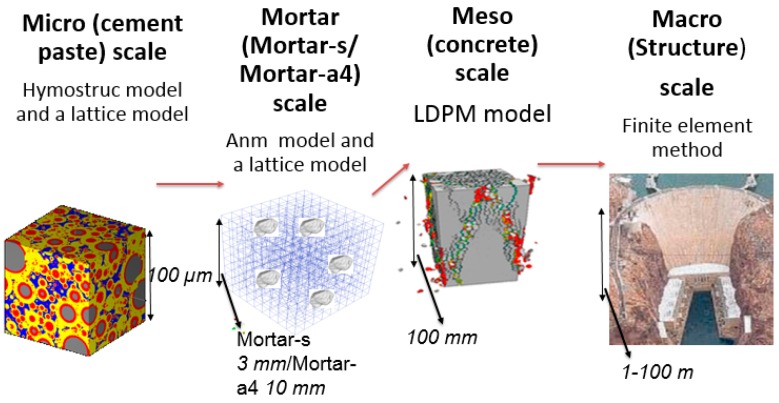
The different scales and models used in the suggested multiscale analysis.

The various scales and models used in this study are depicted in Figure 1. The cement paste scale is characterized by cement products prior to and following the hydration process; the cement grain particle size range is 1–50 μm. For this scale, we suggest using the HYMOSTRUC3D and lattice models. The mortar-s scale includes the cement paste as a matrix, sand, and an Interface Transition Zone (ITZ); the sand particle size range is 1.2–0.5 mm. For this scale, we suggest using the Anm and lattice models. The mortar-a4 scale includes the combination of mortar-s as a matrix, with aggregates smaller than 4 mm and an interface layer between them; the aggregate size range is 2.36–4 mm. The designation mortar-a4 was given to emphasize the fact that the aggregates are smaller than 4 mm. For this scale, we suggest using the Anm and lattice models. The concrete scale includes aggregates larger than 4 mm (and usually up to 20 mm) and mortar-a 4 as a matrix; we consider this scale to be the concrete scale. For this scale, we suggest using the LDPM. The structure scale is usually solved by using the Finite Element Method (FEM) and is beyond the scope of this study. The size of the unit cells in each scale was determined by a numerical study, which compares different sizes and mesh refinements [38].

Finally, a validation process is presented by the comparison of the macro scale results to a uniaxial compression test. Although a macroscopic validation study of this procedure is presented here, it is recommended that future research includes a comparison to additional experiments in each scale.

## 2. Methodology of Research

**Figure 2:**
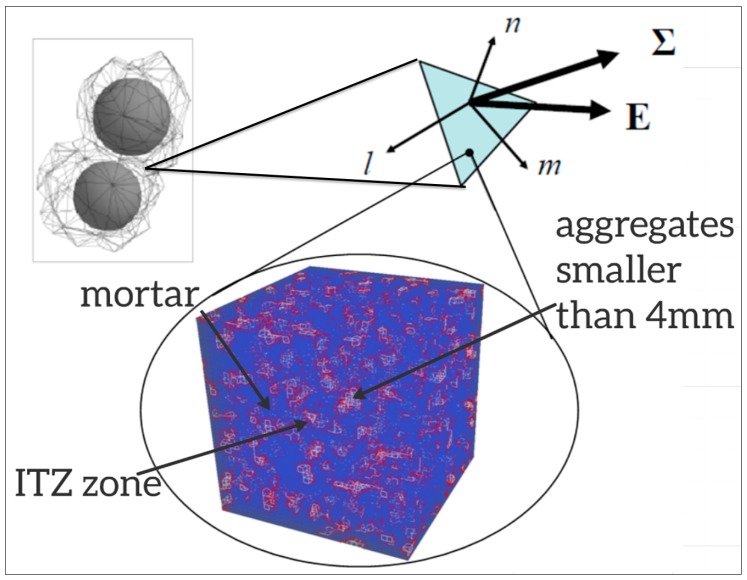
The location of the LDPM facet and its unit cell (mortar in Blue, ITZ in Red, and aggregates smaller than 4 mm in White).

The upscaling procedure presented here bridges the scales by deriving the elastic, tensile, and shear parameters required for the LDPM from the characteristics of the lower scale. Each LDPM parameter relates to a different failure mode and can, therefore, be derived from a different set of simulations. The constitutive equations of the LDPM represent the mechanical behavior at the facets in which the mortar is located; therefore, it can be assumed that the facet failure modes can be characterized by a unit cell of the mortar, which includes aggregates smaller than 4 mm; this is shown in Figure 2, which depicts the location of the facet between two aggregates and the mortar-a4 unit cell, which represents its mechanical behavior. 

**Figure 3:**
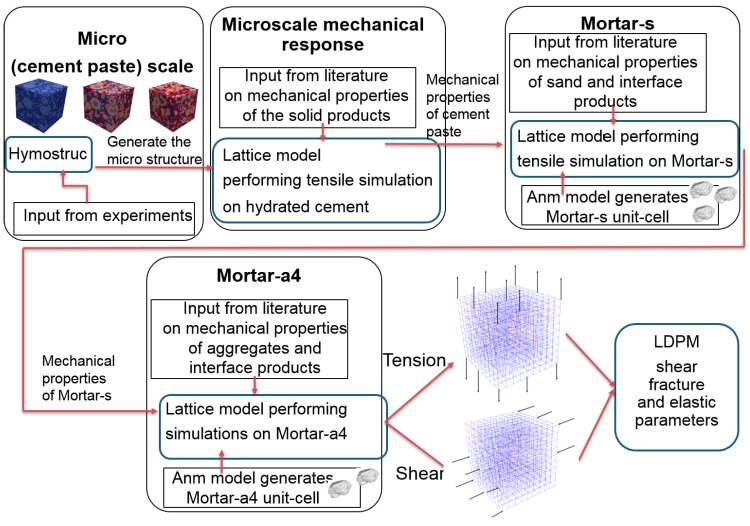
Flowchart of the suggested upscaling procedure.

The suggested methodology involves chemical simulation, mechanical simulation of the hydrated cement, mechanical simulation of the mortar-s unit cell, and mechanical simulation of the mortar-a4 unit cell, which provide the parameters used as inputs into the LDPM. A flowchart of the suggested methodology is illustrated in Figure 3. The mineralogical and chemical composition of the cement is required as input to the HYMOSTRUC3D model, and the output of this step is the microstructure of the hydrated cement paste. The microstructure of the cement paste, together with the mechanical properties of the microstructure constituents, are then used to perform a tension simulation using the lattice model [34], which results in an output of the mechanical properties of the cement paste. Next, the mechanical properties of the cement paste are used, together with the mechanical properties of sand, as an input for a lattice model that simulates the mechanical performance of mortar-s under tensile testing. The mechanical properties of the mortar-s, together with the properties of the aggregate, are then used as inputs for a lattice model that simulates the mechanical performance of mortar-a4. In the current study, two simulations (tension and pure shear) of the mortar-a4 unit cell are used to evaluate the elastic, fracture, and shear parameters of the LDPM.

This paper presents a successful upscaling of five LDPM parameters from the lower scales; these are related to the fracture, shear, and elastic modes. The upscaling of the remaining ten parameters requires further study related to the compression microscale model and, therefore, is not presented in this paper. The experimental data needed as input have been collected from [39,40] and include the following information: At the cement paste (microscopic) scale, the chemical composition was obtained from inductively coupled plasma mass spectrometry (ICP); and the mineralogical composition of the cement was obtained using X-ray analysis (XRD). At the macroscopic level, a uniaxial compression test (at the age of 169 days) was performed on a concrete specimen to calibrate and validate the numerical models at the concrete meso scale. It is recommended that future research includes a comparison to additional macroscopic experiments, such as fracture tests, to ensure the uniqueness of the set of the obtained parameters.

The following subsections provide more details related to the LDPM parameters.

### 2.1. Mix Design Parameters

Mix-design parameters are required to create the geometrical definition of the concrete meso structure, including cement content (*c*), water-to-cement ratio (*w*/*c*), aggregate-to-cement ratio (*a*/*c*), maximum aggregate size (*d_a_*), Fuller’s curve coefficient (*n_F_*), and minimum aggregate size (*d*_0_). The first four parameters are obtained directly from the mix design, while the Fuller coefficient is obtained by performing a best fit procedure of the Fuller curve to the sieve analysis experimental data. The parameter *d*_0_ governs the resolution of the model and was chosen, here, to be 4 mm so as to avoid excessive computing demands and to include aggregates in the mortar-a4 scale and take into account failure through the ITZ in the mortar scale, which is more realistic when representing the mechanical behavior of the facet.

### 2.2. Upscaling the LDPM Mechanical Parameters

The LDPM comprises fifteen parameters, which are used in the facet constitutive law. The LDPM is formulated through the relative displacements (and rotations) of adjacent nodes (particles), where, in each facet, there are two directions for shear strains and normal strains. Therefore, different simulation types represent different failure criterions. For convenience, the parameters of the constitutive laws have been divided into the following groups (for more details, see [16]):

#### 2.2.1. Elastic Parameters

**Figure 4:**
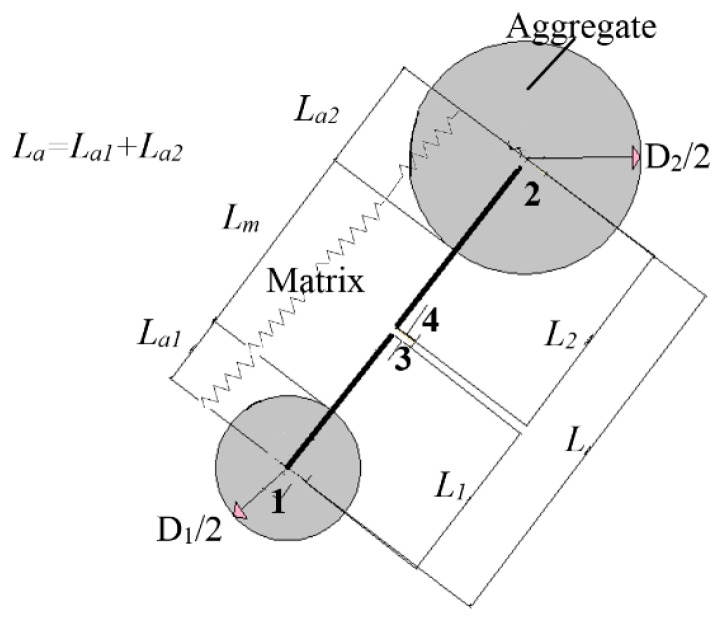
Axial interaction between aggregate centers.

The first group of parameters contains two elastic parameters:

$E_{0}$ is the normal elastic modulus (stiffness of the normal facet behavior), presented in Equation (1), which governs the LDPM response in the elastic mode of operation.

(1) $$\frac{L}{E_{0}}=\frac{L_{a}}{E_{a}}+\frac{L_{m}}{E_{m}}$$

where *E_m_* and *E_a_* are the mortar and aggregate modules of elasticity, respectively; *L_m_* and *L_a_* are the normal length of the mortar and aggregates, respectively; L_a1_ is the radius of aggregate 1; *L_a_*_2_ is the radius of aggregate 2, *L_a_* = *L_a_*_1_ + *L_a_*_2_; and *L* is the distance between two adjacent aggregates (elements), as presented in Figure 4.

*α* is the shear-normal coupling parameter represented in Equation (2)

(2) $$\alpha =\frac{1-4\nu }{1+\nu }$$

where ν is the assumed Poisson’s ratio parameter of concrete.

**Figure 5:**
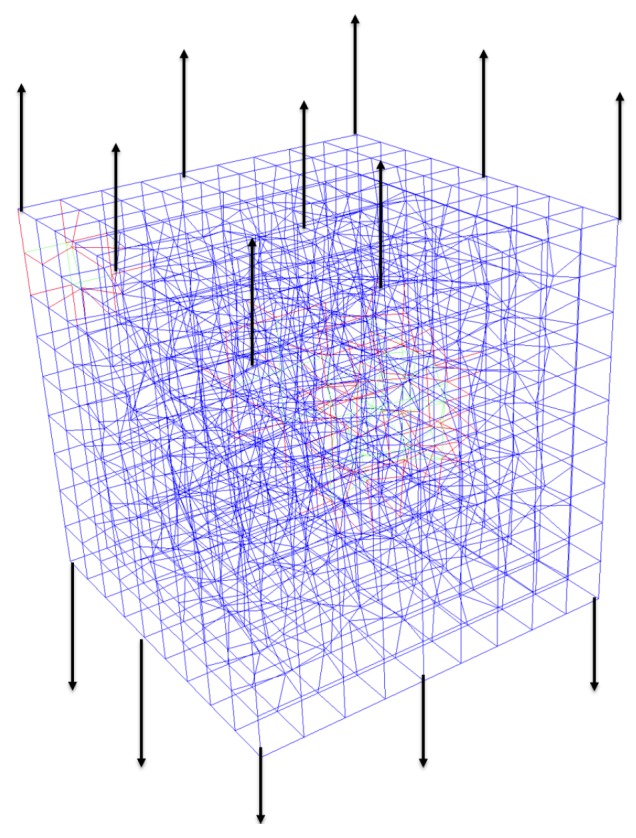
Uniaxial tension numerical test based on the lattice model.

In this paper, the upscaling of these parameters is suggested. The upscaling from a numerical uniaxial tension simulation of the mortar-a4 scale is achieved using the lattice model, as suggested by [41] and shown in Figure 5.

The Young’s modulus of the mortar matrix (*E_m_*) is obtained from the slope of the stress–strain curve, which is obtained from the tensile simulation.

#### 2.2.2. Fracture Parameters

The second group of parameters contains two parameters, which represent the tensile forces between the aggregates at a facet point (normal direction). The parameters are:

$\sigma_{t}$, tensile strength; and $l_{t}$, modified characteristic length for concrete using the LDPM (for more details see [16,42]), as depicted in Equation (3).

(3) $$l_{t}=\frac{2E_{o}G_{t}}{\sigma_{t}^{2}}$$

The tensile strength and fracture energy have been upscaled from a uniaxial tension simulation of the mortar-a4, as shown in Figure 5. The peak value at the stress–strain curve is the tensile strength, and the fracture energy is calculated from the area under the stress–strain curve. The characteristic length is evaluated from Equation (3).

#### 2.2.3. Compressive Parameters

The third group of parameters contains six parameters, which represent a pore collapse and material compaction behavior. The upscaling of this group of parameters is not shown in this paper.

#### 2.2.4. Friction Parameters

The fourth group of parameters contains three parameters, which represent the shear forces between the aggregates at the facet due to frictional effects. The upscaling of this group of parameters is not shown in this paper.

#### 2.2.5. Shear Parameters

The fifth group of parameters contains two parameters, which represent the interaction between shear and tensile behavior.

$n_{t}$ is the softening exponent, which governs the interaction between shear and tensile behavior during softening at the facet level. The upscaling of this parameter is not shown in this paper.

σsσt is the shear-to-tensile strength ratio, where $\sigma_{s}$ is the facet strength for pure shear.

**Figure 6:**
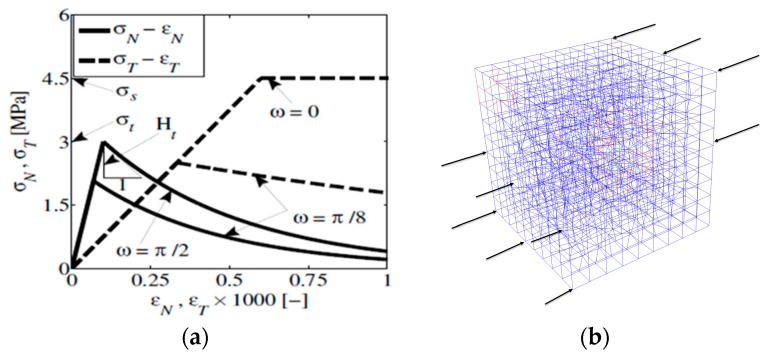
(**a**) Typical stress–strain curves at the LDPM facet according to Cusatis [16]; and (**b**) pure shear lattice model.

In this study, the shear strength parameter is evaluated by performing a simulation test on the mortar-a4 with shear load. Figure 6a describes typical tension and shear stress–strain curves followed by failure behavior, as reported by Cusatis [16], where $\sigma_{T}$ is the shear stress, $\varepsilon_{T}$ is the shear strain, $\sigma_{N}$ is the normal stress, $\varepsilon_{N}$ is the normal strain, and *ω* represents the degree of interaction between shear and normal loading, such that *ω* = 0 represents pure shear and *ω* = *π*/2 represents pure tension. Figure 6b presents a pure shear simulation needed to obtain the value of the shear strength. 

The pure shear test is suitable to describe the failure criterion theory of the LDPM, as the discrete compatibility equations (strain vs. displacements) are formulated through the relative displacements of adjacent nodes (particles), where, in each facet, there are two directions for shear strains. 

The $\sigma_{s}$ parameter is estimated from the peak value of the shear stress–shear strain curve. From the simulation results of pure shear and pure tension, the σsσt shear-to-tensile strength ratio is calculated. 

## 3. Application of the Suggested Upscaling Methodology 

### 3.1. Microstructure Modelling of the Cement Paste

**Figure 7:**
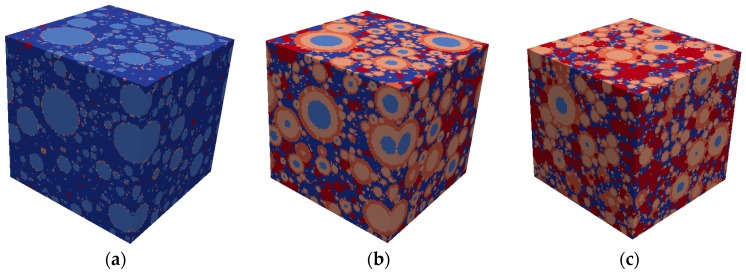
Simulated microstructures of cement paste for specimen size 100 μm × 100 μm × 100 μm: At (**a**) 3 h; (**b**) 28 d; and (**c**) 169 d (porous in dark blue, unhydrated cement in bright blue, inner hydration product in bright orange, outer hydration product in dark orange, and CH-calcium hydroxides in red).

In this study, the HYMOSTRUC3 model [28], which has been validated through experiments (e.g., [43]), is used to simulate the cement hydration and microstructure of the cement paste. The simulations were performed for a specific concrete mix design [39,40]. The specimen size simulated to obtain the microstructure of cement is 100 μm × 100 μm × 100 μm, with the mix as follows: Portland cement with a maximum cement size of 50 μm, a *w*/*c* ratio of 0.567, air content 0.6%, and a curing temperature of 20 °C. The mineralogical compositions of the cement were obtained using XRD analysis [40], as summarized in Table 1. The microstructures at the curing age of 3 h, 28 d (672 h), and 169 d (4056 h) are shown in Figure 7. The simulation results for 169 d were used since the macroscopic experiments were performed at that age.

### 3.2. Evaluation of the Mechanical Properties of the Cement Paste 

A three-dimensional lattice fracture model [34] was used to evaluate the mechanical properties of the cement paste at the age of 169 d. Verification and validation (including an experimental study) of the suggested lattice model are presented in [44]. This particular age of the cement paste was chosen for the simulations so as to be consistent with the age of the concrete in the specimens that were used during the uniaxial compression test at the macroscopic scale. The mechanical properties of each constituent of the microstructure are presented in Table 2 (see also [41]). At this scale, we suggest a two-step homogenization procedure, as follows:

First homogenization step: The microstructure obtained from the HYMOSTRUC3 simulation at the age of 169 d is decomposed to 1000 cubical sub-specimens, with a size of 10 μm × 10 μm × 10 μm. Each sub-specimen is simulated using a three-dimensional lattice fracture model and analyzed to obtain its stress–strain curve. The boundary conditions of the uniaxial tensile simulation are fully constrained on the faces normal to the longitudinal direction and free on the other faces. The loading is imposed incrementally in the longitudinal direction.

The spherical particles, representing the microstructure of hydrated cement, are converted to a voxel-based digital image by applying the ImgLat lattice construction method [41]. In our study, the chosen resolution was 1 μm/voxel. In every voxel cell there is a sub-cell, and a random node is created in each sub-cell to represent the heterogeneity of the material. The value of randomness is in the range of 0–1 and controls the degree of heterogeneity, where 1 is the maximum heterogeneity, as the sub-cell becomes identical to the cell size and the materials have the maximum degree of disorder. The chosen randomness parameter was 0.5 [41]. The adjacent nodes were connected with Timoshenko beam elements forming a lattice construction. The beam elements have a circular cross section with an area equivalent to a voxel cell of 1 μm × 1 μm. Different mechanical properties are assigned for each beam element, depending on the location of its two nodes in the microstructure. If the location of the two nodes that connect the beam element are in the same phase, then the element is assigned with the same property; otherwise, the element is assigned with an interface element that depends on its geometry. The composition of the microstructure is characterized by five different phases, i.e., unhydrated cement, inner products, outer products and CH. In addition, interface material properties are assigned between the phases, while the pores are represented by a void (i.e., removing the beam element related to the pores). In this manner, nine material types are generated, as presented in Table 2 (in which the properties were calibrated to cement Portland concrete [41]). Therefore, we assume that the hydration products have the same mechanical properties. However, if other cement types are used, these parameters must be measured and validated.

The mechanical properties that are required for the lattice model are the Young modulus, shear modulus, tensile strength, and compression strength, all obtained from [41]. The experimental results for the elastic properties of solid phases are measured and summarized in [45]. The tensile strength ratio of each phase is considered equal to the nanoindentation hardness ratio, as discussed in [46]. The compressive strength is an approximation, and equals to ten times the tensile strength.

**Figure 8:**
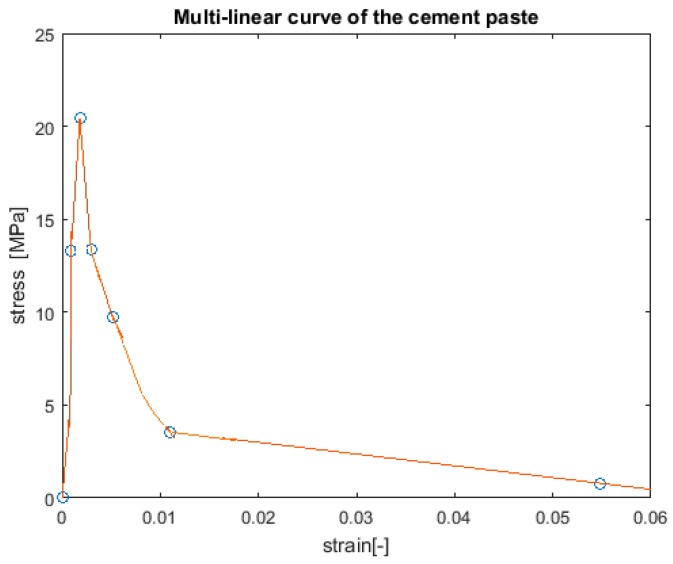
Cement paste stress–strain curve.

Second homogenization step: Here, the cement paste unit cell, at the size of 100 μm × 100 μm × 100 μm, is analyzed using the results from the first homogenization step. The result of each of the 1000 sub-specimens is a stress–strain curve represented by a multilinear curve. These multilinear curves describe the local mechanical properties of the beam elements of the cement paste lattice model of the unit cell. A uniaxial tension simulation of the cement paste unit cell provides the homogenized mechanical properties, which, in turn, represent the matrix at the mortar-s scale model, as depicted in Figure 8.

### 3.3. Upscaling the Mortar-S Scale

The mortar-s scale includes the cement paste as a matrix, sand as inclusions, and interface transition zones. The mechanical properties of the sand, and the interface between the sand and the cement paste, are presented in Table 3. The mechanical properties of the cement paste are described as multilinear curves obtained from the results in Section 3.2 (see Figure 8). It is important to note that the gradients in material properties resulting from the different hydration stages in the specimen are neglected (see, for instance, [47,48]). Uniaxial tensile simulation was constrained by a high-friction (HF) boundary condition, so that lateral displacement and rotations have been prevented on the faces, which are normal to the loads, while the other faces are free to displace or rotate. To allow a reasonable computational time and maintain the accuracy of the results, the specimen size of this scale was chosen to be 3 mm × 3 mm × 3 mm, which is 2.5 times the maximum particle size for the mortar-s scale and is considered as homogeneous, as recommended in [38]. The size of the voxel in the mortar-s model needs to be equal or smaller than the cement paste unit cell size; therefore, the chosen resolution was 0.1 mm/voxel (see [41] for more information). The unit cell of the mortar-s scale was obtained using the Anm material model, which is used to allocate irregular shaped sand particles in the unit cell according to periodic morphology [33]. For this purpose, the Amn model parameters and the sieve distribution properties of the sand are presented in Table 3 and Table 4, respectively.

**Figure 9:**
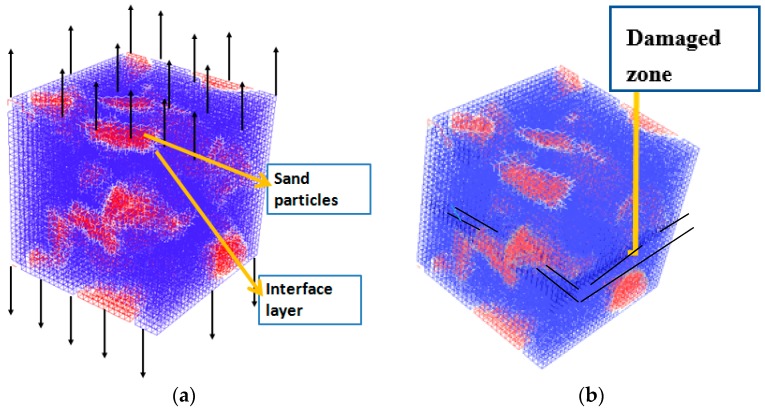
(**a**) Uniaxial tension analysis on the mortar-s scale (cement paste in blue, sand particles in red, and interface layer in white); and (**b**) the resulting damaged zone on mortar-s scale (damaged zone is underlined in black).

The lattice model [34] is again used to simulate the mortar-s unit cell that was obtained using the Anm model (Figure 9). The input parameters, including the mechanical properties of the cement paste (Figure 8), the sand, and the interface mechanical properties, are presented in Table 5 (see also [41]).

### 3.4. Upscaling the Mortar-a4 Scale

**Figure 10:**
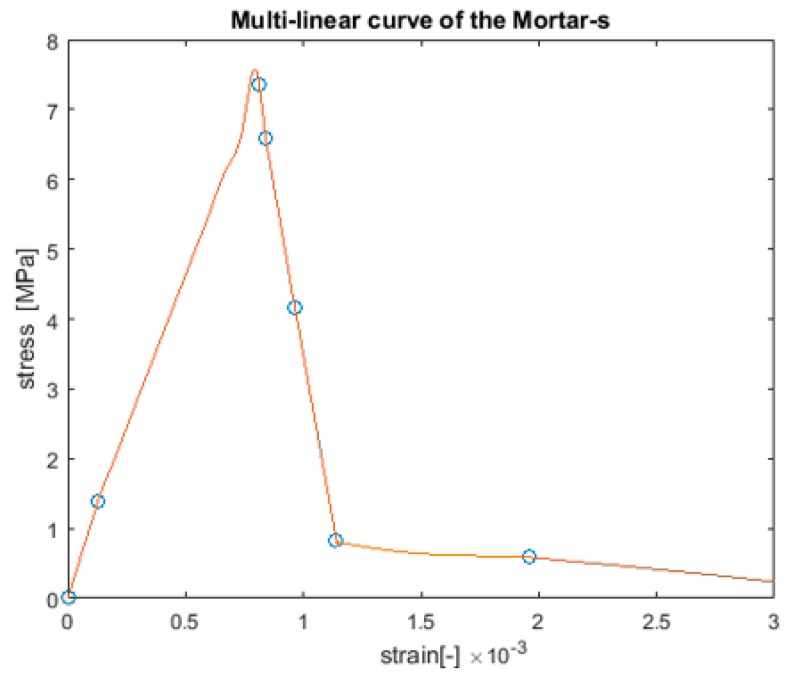
Mortar-s stress–strain curve.

This scale represents the mortar-a4, which contains the matrix (mortar-s), aggregates smaller than 4 mm, and an interface layer between them. The Young modulus, shear modulus, tensile strength, and compression strength of the mortar-s mechanical properties are obtained from the results of the lower scale (Figure 10). The unit cell size chosen for this scale is 10 mm × 10 mm × 10 mm and the resolution chosen is 1 mm/Voxel. The Anm model has been applied to obtain the unit cell geometry; it creates the irregular shape aggregate particles and places them in the unit cell with periodic morphology along the unit cell boundaries (for more details, see [33]). The Amn model parameters and the sieve distribution properties of the sand are presented in Table 6 and Table 7, respectively. The mechanical properties of the aggregates and the interface between the aggregates are given in Table 8.

The lattice model [34] is again used to simulate the mortar-a4 unit cell that was obtained using the Anm model. The input parameters, including the mechanical properties of the mortar-s (Figure 10), the aggregates, and the interface mechanical properties are presented in Table 8 (see also [41]).

### 3.5. Evaluation of the LDPM Elastic, Fracture, and Shear Parameters

**Figure 11:**
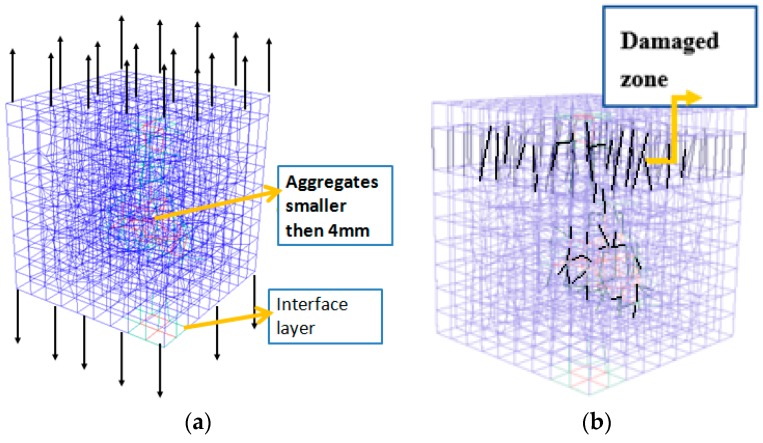
(**a**) Uniaxial tension test on the mortar-a4 scale (mortar-s in blue, aggregate particles in red, and interface layer in turquoise); and (**b**) the resulting damaged zone for the uniaxial tension simulation (damaged elements in black).

A set of simulations was performed at the mortar-a4 scale to evaluate the elastic ($E_{0}$, v), fracture ($l_{t}$, $\sigma_{t}$), and shear ($\sigma_{t}$/$\sigma_{t}$) parameters for the LDPM. The simulations that must be performed to evaluate these LDPM parameters are the uniaxial tensile test and the pure shear test. The tensile simulation is performed by incrementally applying a displacement on the specimen in the longitudinal direction. As mentioned in Section 3.4, the mortar-a4 unit cell size is 10 mm × 10 mm × 10 mm, while the resolution chosen is 1 mm/Voxel. The lattice model and crack pattern of the mortar-a4 unit cell for the uniaxial tension simulation are presented in Figure 11. 

**Figure 12:**
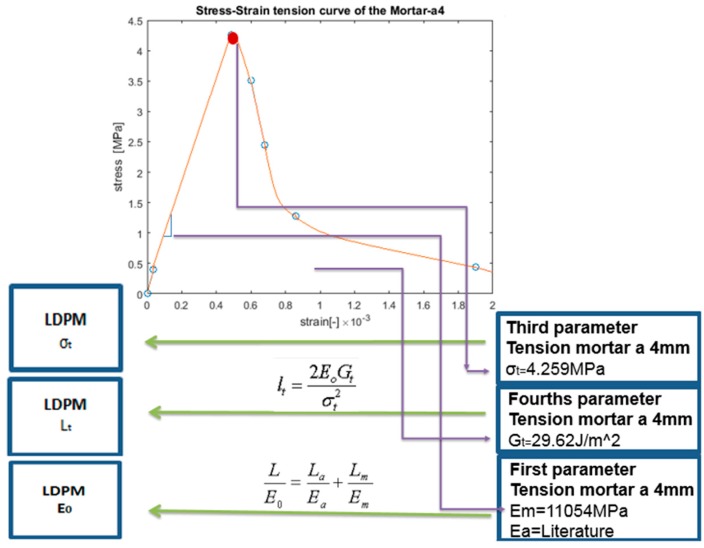
Mortar-a4 stress–strain curve.

The stress–strain curves obtained from the tension simulation and the upscaling parameters are presented in Figure 12. The peak value in Figure 12 is the tensile strength, $\sigma_{t}$. The fracture energy, *G_t_*, can be evaluated from the area under the stress–strain curve and the LDPM characteristic length is defined as $l_{t}=\frac{2E_{o}G_{t}}{\sigma_{t}^{2}}$. The normal elastic modulus is calculated for each facet using Equation (1). The module elasticity of the mortar is evaluated from the slope of the linear part of the mortar-a4 stress–strain curve (Figure 12). The Poisson ratio of the concrete is assumed to be 0.15 and the LDPM *α* parameter is calculated using Equation (2).

**Figure 13:**
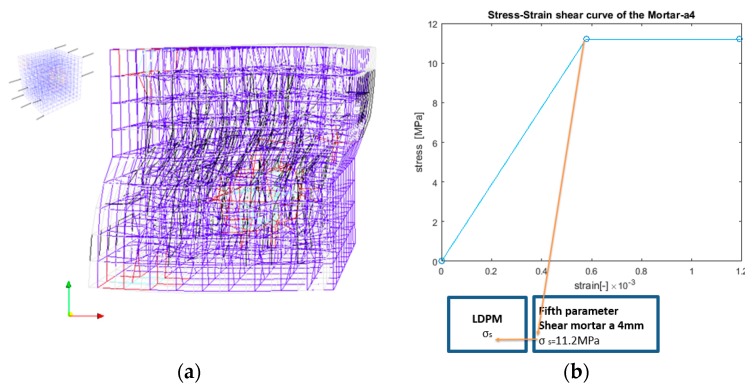
(**a**) Shear damage mode (damaged elements in blue; white box represents the non-deformed shape); and (**b**) mortar-a4 shear stress–shear strain curve.

The shear simulation was loaded by prescribing the horizontal displacement to the left and right edges, as presented in Figure 6b. On the faces that were loaded (with incremental displacement), the nodes were restrained against displacement, while the rotations were free. The shear stress–shear strain shown in Figure 13b and the crack pattern shown in Figure 13a were obtained from the simulation of the pure shear test. The shear strength parameter was obtained as the peak value of the shear stress–shear strain curve and the LDPM σsσt was evaluated using this value. 

## 4. Results

**Figure 14:**
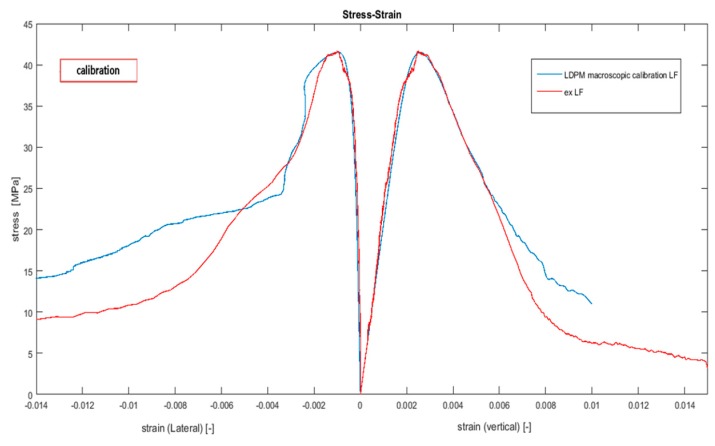
Uniaxial compression test experimental and numerical results using macroscopic calibration (the blue line represents the average of three different particle placements of the LDPM numerical simulation, and the red curve represents the average of three different specimen tests).

In this section, a macroscopic fit (Figure 14) was compared to the suggested upscaled strategy. The LDPM comprises two sets of parameters, as described in Section 2. The mix-design parameters are described in Section 2.1 and the mechanical parameters are described in Section 2.2. The LDPM has been widely validated for the analysis of concrete specimens using a comparison to experimental results (e.g., [16,17,39,40]). In our study, we calibrated the LDPM mechanical parameters by using two methods. First, the calibration of the 15 LDPM parameters was achieved by comparing the macroscopic numerical results with the macroscopic experimental results (see [39,40] and Figure 14). Second, the elastic, fracture, and shear parameters were evaluated by using the suggested upscaling methodology, while the remainder of the LDPM parameters were estimated by using a curve-fitting procedure. The calibration process, depicted in Figure 14, commenced by assuming initial values based on the work of [17] and changing the parameters to fit the curve. The macroscopic tests and the simulation were conducted on a 100 mm specimen size, and were restricted to low friction boundary conditions (*μ*_s_ = 0.03, *μ*_d_ = 0.0084, s_0_ = 0.0195 mm) between the loading plates and the concrete specimen. HF conditions gives rise to the stress peak value, which is higher than the low friction (LF) conditions, due to the shear stress between the loading platen and the specimen. The post-peak slope of the stress–strain curve is also strongly dependent on the type of boundary restraint; a steeper slope under LF conditions is associated with a lower ductility. In the case of HF tests, parts of the specimen were confined, with the result that these areas are less likely to fail. As a result, the fracturing is forced to occur in the unconfined parts of the specimen. Selecting LF conditions results in a lower peak value and a steeper slope at the post-peak region; however, the numerical simulation and the experiments are compared under the same conditions. In addition, the LF experimental results have a narrow scattering profile, and, therefore, we prefer to use them for the calibration process. In [17], one type of experiment was used to calibrate the parameters, but various other types of experiments are required to validate the model, as has been done in [16,17,39,40]. The calibrated results are presented in Figure 14.

**Figure 15:**
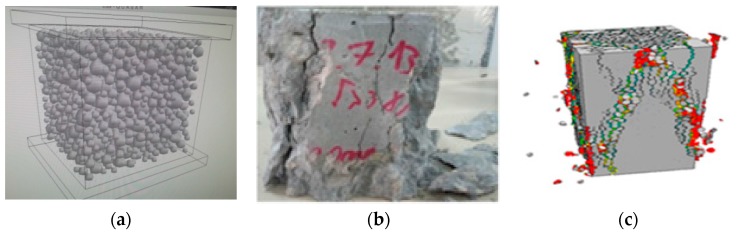
(**a**) Patical size distribution of the LDPM; (**b**) experimental crack pattern for the uniaxial test; and (**c**) crack pattern obtained by the LDPM simulation.

The experimental crack pattern is presented in Figure 15b, the crack pattern obtained by the LDPM simulation is presented in Figure 15c, and a scheme of the particle size distribution of aggregates is presented in Figure 15a.

The parameters that govern the generation of concrete meso structures are presented in Table 9, as reported in [39,40], and the macroscopic-calibrated LDPM mechanical parameters are presented in Table 10.

To validate the five parameters that were upscaled using the suggested methodology, we replaced the appropriate values, shown in Table 10, and present the new values in Table 11. The remaining ten parameters that appear in Table 12 were calibrated again using curve fitting. 

**Figure 16:**
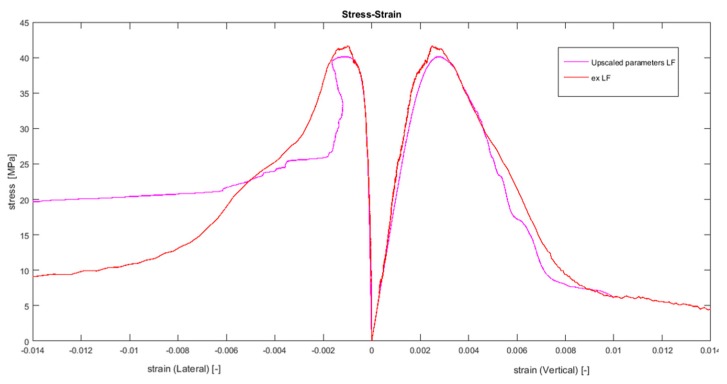
Uniaxial compression test; experimental and numerical results using upscaled parameters.

A comparison of the values in Table 11 to the appropriate value in Table 10, which were obtained by two very different methods, shows an excellent fit. It is important to note that further experiments, such as fracture tests, are required to ensure the uniqueness of the set of parameters that were obtained by using the suggested inverse analysis. Note that the normal elastic modulus, $E_{o}$, for each facet was evaluated separately in this paper and, therefore, it cannot be compared to the macroscopic value. The aggregate module of elasticity was chosen from a range of values, as published in [47]. This value was calibrated according to the experimental results. Since the aggregate module of elasticity is higher than expected, at this stage of the research, we suggest using the value of $E_{o}$ from the macroscopic experiment. Another comparison is presented at the macroscopic scale in Figure 16, which shows the comparison of the macroscopic longitudinal and transverse displacements, which were evaluated by using the upscaled LDPM parameters, to the experimental results. The good fit of the stress–strain curves, presented in Figure 16, demonstrates that the discrepancy of parameters obtained by the different methods is negligible and that the proposed method of bridging the scales is an effective way to provide accurate results.

## 5. Discussion and Conclusions

This study provides a methodology for bridging the cement and concrete scales. Scaling down to the chemical composition of the cement paste can, potentially, illuminate the influence of the lower scales on the concrete macroscopic strength. 

We suggest an upscaling procedure between the cement paste scale and the elastic, fracture, and shear concrete LDPM parameters using existing validated models. The LDPM was chosen to represent the concrete behavior at the mesoscale level due to its ability to capture the concrete macroscopic size and shape effects, and because of its ability to take into account the influence of concrete mix components. The three lower-scale models chosen for the suggested upscaling procedure are the HYMOSTRUC3D model, which was used for the cement hydration analysis to obtain the cement paste microstructure; the Anm model, which was used for the morphology of the mortar unit cells; and a lattice model, based on Timoshenko beam elements, which was used for the mechanical analysis of the cement paste and mortar scales. 

The presented upscaling procedure is expected to reduce the demand of experimental studies for the calibration process of the LDPM. Moreover, the LDPM is expected to be enhanced by including important lower-scale phenomena, which have not previously been considered in this manner. The upscaling procedure was conceptually presented; however, due to lack of experimental data at the different scales, it could not be fully validated.

This study involves the upscaling of the elastic, fracture, and shear LDPM parameters, which suggests new calibration abilities by starting the calibration process with a number of parameters obtained by the multiscale analysis of the lower scales. Calibrating the entire set of 15 LDPM parameters might be a very demanding and challenging task with a high number of variations. Hence, using the suggested methodology, the calibration process can be started with a number of known parameters to improve the effectiveness of the calibration procedure. 

Further research is needed to enable upscaling the full set of LDPM parameters, including the remaining ten parameters that are related to the friction and compression behavior. Generally speaking, the full set of parameters requires hydrostatic and confined compression simulations of the lower scales to obtain the six compression parameters, and combined compression and shear loading simulations of the lower scales to obtain the friction parameters. In addition, a complete calibration procedure should include an experimental study on the lower scale. These procedures should include a hydration test and uniaxial tensile tests for the cement paste and mortar scales.

The result of this research is a powerful methodology that, after further development, will have broad industrial applications. Potential users of the methodology are producers of cement-based materials and structural designers.

## Figures and Tables

**Table 1:** Cement specifications.

Characteristic	Inputs Specification
Mineralogical composition (%)	C3S: 54.9, C2S: 19.1, C3A: 4, C4AF: 8.8
Chemical composition (%)	Al_2_O_3_: 6.41, SiO_2_: 20.39, MgO: 1.08, CaO: 58.58, Fe_2_O_3_: 3.92, SO_3_: 2.92, Na_2_O: 0.22, K_2_O: 0.44
Minimum particle diameter	1 μm
Cement fineness (Rosin–Rammler distribution)	*n* = 1.05771, b = 0.04282
Curing temperature	20 °C

**Table 2:** Specifications of the lattice model inputs used in the simulation of cement paste (from [41]).

No.	Element Type	Young Modulus E (GPa)	Shear Modulus G (GPa)	Tensile Strength $f_{t}$ (GPa)	Compression Strength $f_{c}$ (GPa)
1	Unhydrated cement	135	52	1.8	−18
2	Interface–Unhydrated and Inner	49	20	0.24	−2.4
3	Inner product	30	12	0.24	−2.4
4	Interface–Inner and Outer	25	10	0.15	−1.5
5	Outer product	22	8.9	0.15	−1.5
6	Interface–Outer and CH	26.4	10.6	0.15	−1.5
7	(CH)–Calcium Hydroxides	33	13.2	0.264	−2.64
8	Interface–Unhydrated and Outer	38	15.2	0.15	−1.5
9	Interface–Inner and CH	31.5	12.6	0.24	−2.4

**Table 3:** Mortar-s Anm model input parameters.

Input Category	Input Specification
Specimen size (mm^3^)	3 × 3 × 3
Sand mass (g)	0.04
Sand density (g/mm^3^)	0.00265

**Table 4:** Sieve distribution of the mortar-s scale.

Input Category	Inputs Specification
Sieve distribution (mm)	1.2–0.5
Mass percentage (%)	100

**Table 5:** Sand and interface material properties (from [41]).

No.	Element Type	Young Modulus E (MPa)	Shear Modulus G (MPa)	Tensile Strength $f_{t}$ (MPa)	Compression Strength $f_{c}$ (MPa)
1	Sand	70,000	29,000	24	−240
2	Interface–Sand and Cement	22,000	8900	0.75	−7.5

**Table 6:** Mortar-a4 Anm model input parameters.

Input Category	Input Specification
Specimen size (mm^3^)	10 × 10 × 10
Aggregate mass (g)	0.093
Aggregate density (g/mm^3^)	0.00265

**Table 7:** Sieve distribution of the mortar-a4 scale.

Input Category	Input Specification
Sieve distribution (mm)	2.36–4
Mass percentage (%)	100

**Table 8:** Aggregate and interface material properties [41].

No	Element Type	Young Modulus E (MPa)	Shear Modulus G (MPa)	Tensile Strength $f_{t}$ (MPa)	Compression Strength $f_{c}$ (MPa)
1	Aggregates	120,000	29,000	24	−240
2	Interface–Aggregates and Mortar-s	41,000	17,000	1	−10

**Table 9:** LDPM mix-design parameters.

Symbol	C (Kg/m^3^)	*w*/*c*	*a*/*c*	Do (mm)	Da (mm)	Nf (-)
Sec.	391	0.567	2.2532	4	14	0.425

**Table 10:** LDPM mechanical parameters.

**E_0_ (Mpa)**	***α***	***σ*_t_ (Mpa)**	**L_t_ (mm)**	***σ*_s_/*σ*_t_**	**n_t_**	***σ*_co_ (Mpa)**	**H_co_/E_0_**
30150	0.38	4.03	120	2.7	0.5	70	0.4
**K_c0_**	**K_c1_**	**K_c2_**	***μ*_o_**	***μ*_∞_**	***σ*_No_**	**H_d_/E_0_**	
2	1	5	0.2	0	600	1	

**Table 11:** LDPM upscaled parameters.

Parameters	$E_{o}[MPa]=\frac{1}{1-2\nu }E$ (LDPM Equation)	α[−]=1−4ν1+ν	*σ*_t_ [MPa]	G_t_ = L_t_*σ*_t_^2^/2E_0_ [N/m]	*σ*_s_/*σ*_t_
LDPM calibrated macroscopically parameters	30,150	0.38	4.03	32.32	2.7
Evaluated parameters from the lower scale	$\begin{array}{l} E_{m}=11054\\ E_{a}=120000 \end{array}$	0.35	4.259	29.62	2.63

**Table 12:** Values of the remaining ten LDPM parameters.

n_t_	*σ*_co_ (Mpa)	H_co_/E_0_	K_c0_	K_c1_	K_c2_	*μ*_o_	*μ*_∞_	*σ*_No_	H_d_/E_0_
0.5	200	0.4	2	1	5	0.2	0	600	1

## References

[1] Riedel W., Thoma K., Hiermaier S., Schmolinske E. Penetration of reinforced concrete by BETA-B-500 numerical analysis using a new macroscopic concrete model for hydrocodes. Proceedings of the 9th International Symposium on the Effects of Munitions with Structures.

[2] Chengqing L. (2008). Application of Damaged Plasticity Model for Concrete. Struct. Eng..

[3] Fang Q., Huan Y., Zhang Y.-D., Chen L. (2007). Investigation into static properties of damaged plasticity model for concrete in ABAQUS. J. PLA Univ. Sci. Technol. (Nat. Sci. Ed.).

[4] Munjiza A., Andrews K., White J. (1999). Combined single and smeared crack model in combined finite-discrete element analysis. Int. J. Numer. Methods Eng..

[5] Dahlblom O., Ottosen N.S. (1990). Smeared crack analysis using generalized fictitious crack model. J. Eng. Mech..

[6] Lubliner J. (2008). Plasticity Theory.

[7] Pijaudier-Cabot G., Bažant Z.P. (1987). Nonlocal damage theory. J. Eng. Mech..

[8] Al-Rub R.K.A., Voyiadjis G.Z. (2003). On the coupling of anisotropic damage and plasticity models for ductile materials. Int. J. Solids Struct..

[9] Cusatis G., Bažant Z.P., Cedolin L. (2003). Confinement-shear lattice model for concrete damage in tension and compression: I. Theory. J. Eng. Mech..

[10] Cusatis G., Bazant Z.P., Cedolin L. 3D Lattice model for dynamic simulations of creep, fracturing and rate effect in concrete. Proceedings of the 6th International Conference on Cambridge.

[11] Cusatis G., Polli M., Cedolin L. Mesolevel analysis of fracture tests for concrete. Proceedings of the Fifth International Conference on Fracture Mechanics of Concrete and Concrete Structures—FraMCoS-5, Vail Cascade Resort.

[12] Cusatis G., Cedolin L. (2007). Two-scale study of concrete fracturing behavior. Eng. Fract. Mech..

[13] Cusatis G., Bažant Z.P., Cedolin L. (2006). Confinement-shear lattice CSL model for fracture propagation in concrete. Comput. Methods Appl. Mech. Eng..

[14] Cusatis G., Pelessone D., Mencarelli A., Baylot J.T. Simulation of reinforced concrete structures under blast and penetration through lattice discrete particle modeling. Proceedings of the ASME 2007 International Mechanical Engineering Congress and Exposition.

[15] Cusatis G., Bažant Z.P., Cedolin L. (2003). Confinement-shear lattice model for concrete damage in tension and compression: II. Computation and validation. J. Eng. Mech..

[16] Cusatis G., Pelessone D., Mencarelli A. (2011). Lattice discrete particle model (LDPM) for failure behavior of concrete. I: Theory. Cem. Concr. Compos..

[17] Cusatis G., Mencarelli A., Pelessone D., Baylot J. (2011). Lattice discrete particle model (LDPM) for failure behavior of concrete. II: Calibration and validation. Cem. Concr. Compos..

[18] Cusatis G., Mencarelli A., Pelessone D., Baylot J.T. Lattice discrete particle model (LDPM) for fracture dynamics and rate effect in concrete. Proceedings of the Structures Congress 2008: 18th Analysis and Computation Specialty Conference.

[19] Van Mier J.G. (2012). Concrete Fracture: A Multiscale Approach.

[20] Etse G., Caggiano A., Vrech S. (2012). Multiscale failure analysis of fiber reinforced concrete based on a discrete crack model. Int. J. Fract..

[21] Nguyen V.P., Stroeven M., Sluys L.J. (2012). Multiscale failure modeling of concrete: Micromechanical modeling, discontinuous homogenization and parallel computations. Comput. Methods Appl. Mech. Eng..

[22] Montero-Chacón F., Marín-Montín J., Medina F. (2014). Mesomechanical characterization of porosity in cementitious composites by means of a voxel-based finite element model. Comput. Mater. Sci..

[23] Gal E., Ganz A., Hadad L., Kryvoruk R. (2008). Development of a concrete unit cell. Int. J. Multiscale Comput. Eng..

[24] Gal E., Suday E., Waisman H. (2013). Homogenization of materials having inclusions surrounded by layers modeled by the extended finite element method. Int. J. Multiscale Comput. Eng..

[25] Gal E., Kryvoruk R. (2011). Fiber reinforced concrete properties—A multiscale approach. Comput. Concr..

[26] Grigorovitch M., Gal E. (2014). A new method for calculating local response in elastic media—The embedded unit cell approach. Comput. Model. Concr. Struct..

[27] Flannery B.P., Deckman H.W., Roberge W.G., D’Amico K.L. (1987). Three-dimensional X-ray microtomography. Science.

[28] Ye G., Van Breugel K., Fraaij A. (2003). Three-dimensional microstructure analysis of numerically simulated cementitious materials. Cem. Concr. Res..

[29] Koster M., Hannawald J., Brameshuber W. (2006). Simulation of water permeability and water vapor diffusion through hardened cement paste. Comput. Mech..

[30] Van Breugel K. (1995). Numerical simulation of hydration and microstructural development in hardening cement-based materials (I) theory. Cem. Concr. Res..

[31] Bentz D.P. (2000). CEMHYD3D: A Three-Dimensional Cement Hydration and Microstructure Development Modelling Package.

[32] Bishnoi S., Scrivener K.L. (2009). μic: A new platform for modelling the hydration of cements. Cem. Concr. Res..

[33] Qian Z., Garboczi E., Ye G., Schlangen E. (2016). Anm: A geometrical model for the composite structure of mortar and concrete using real-shape particles. Mater. Struct..

[34] Qian Z., Schlangen E., Ye G., Van Breugel K. Multiscale lattice fracture model for cement-based materials. Proceedings of the ICCM 2012: 4th International Conference on Computational Methods.

[35] RosIN P. (1933). The laws governing the fineness of powdered coal. J. Inst. Fuel..

[36] Ukrainczyk N., Koenders E., Van Breugel K. Multicomponent modelling of Portland cement hydration reactions. Proceedings of the Second International Conference on Microstructural-Related Durability of Cementitious Composites.

[37] Stroeven P. (2000). A stereological approach to roughness of fracture surfaces and tortuosity of transport paths in concrete. Cem. Concr. Compos..

[38] Garboczi E.J., Bentz D.P. (1999). Computer simulation and percolation theory applied to concrete. Annual Reviews of Computational Physics VII.

[39] Sherzer G. Development, Calibration & Validation of Lateral Displacement for Concrete Uniaxial Compression Test. Proceedings of the 10th International Conference on Mechanics and Physics of Creep, Shrinkage, and Durability of Concrete and Concrete Structures.

[40] Sherzer G. (2014). Development, Calibration & Validation of Lateral Displacement for Concrete Uniaxial Compression Test. Master’s Thesis.

[41] Qian Z. (2012). Multiscale Modeling of Fracture Processes in Cementitious Materials. Ph.D. Thesis.

[42] Bažant Z.P., Pijaudier-Cabot G. (1989). Measurement of characteristic length of nonlocal continuum. J. Eng. Mech..

[43] Ye G. (2003). Experimental Study and Numerical Simulation of the Development of the Microstructure and Permeability of Cementitious Materials. Ph.D. Thesis.

[44] Schlangen E., Van Mier J. (1992). Experimental and numerical analysis of micromechanisms of fracture of cement-based composites. Cem. Concr. Compos..

[45] Manzano H., Dolado J., Ayuela A. (2009). Elastic properties of the main species present in Portland cement pastes. Acta Mater..

[46] Qian Z., Schlangen E. (2017). Multiscale modeling framework for fracture in cement-based materials. Int. J. Fract..

[47] Di Luzio G., Cusatis G. (2013). Solidification-microprestress–microplane (SMM) theory for concrete at early age: Theory, validation and application. Int. J. Solids Struct..

[48] Wan L., Wendner R., Liang B., Cusatis G. (2016). Analysis of the Behavior of Ultra High Performance Concrete at Early Age. J. Cem. Concr. Compos..

